# Challenges in defining an optimal approach to formula-based allocations of public health funds in the United States

**DOI:** 10.1186/1471-2458-7-44

**Published:** 2007-03-29

**Authors:** James W Buehler, David R Holtgrave

**Affiliations:** 1 Department of Epidemiology and Center for Public Health Preparedness and Research, Rollins School of Public Health, Emory University, Atlanta, Georgia, USA; 2 Department of Health, Behavior and Society, Bloomberg School of Public Health, Johns Hopkins University, Baltimore, Maryland, USA

## Abstract

**Background:**

Controversy and debate can arise whenever public health agencies determine how program funds should be allocated among constituent jurisdictions. Two common strategies for making such allocations are expert review of competitive applications and the use of funding formulas. Despite widespread use of funding formulas by public health agencies in the United States, formula allocation strategies in public health have been subject to relatively little formal scrutiny, with the notable exception of the attention focused on formula funding of HIV care programs. To inform debates and deliberations in the selection of a formula-based approach, we summarize key challenges to formula-based funding, based on prior reviews of federal programs in the United States.

**Discussion:**

The primary challenge lies in identifying data sources and formula calculation methods that both reflect and serve program objectives, with or without adjustments for variations in the cost of delivering services, the availability of local resources, capacity, or performance. Simplicity and transparency are major advantages of formula-based allocations, but these advantages can be offset if formula-based allocations are perceived to under- or over-fund some jurisdictions, which may result from how guaranteed minimum funding levels are set or from "hold-harmless" provisions intended to blunt the effects of changes in formula design or random variations in source data. While fairness is considered an advantage of formula-based allocations, the design of a formula may implicitly reflect unquestioned values concerning equity versus equivalence in setting funding policies. Whether or how past or projected trends are taken into account can also have substantial impacts on allocations.

**Summary:**

Insufficient attention has been focused on how the approach to designing funding formulas in public health should differ for treatment or service versus prevention programs. Further evaluations of formula-based versus competitive allocation methods are needed to promote the optimal use of public health funds. In the meantime, those who use formula-based strategies to allocate funds should be familiar with the nuances of this approach.

## Background

In 2004, the legislature in state of Louisiana proposed legislation that would mandate the following approach for the regional allocation of funds for public health services related to addictive disorders, developmental disabilities, and mental health. In the proposed legislation, program managers were charged to:

[in the first year of implementation] calculate formulas to adjust raw population data (by age) to account for variations in the incidence/prevalence of disabilities and to adjust for variations in estimated demand for public sector services within...jurisdictions...The formulas shall be based on the most recent reliable scientific information available related to prevalence and demand for services and revised and updated as new research data becomes available... [and in the second year of implementation to] develop budget allocation formulas that incorporate population adjusted per capita funding levels, service access, and service utilization patterns.... The funding formula may also take in account historical funding levels, urban/rural differences in service delivery models and costs, funds reserved for state level administration and planning and state discretionary grants, and such other factors as determined applicable by the offices. When inequities in resource deployment or service access are identified in the regional profiles, then the allocation formulas must include incremental strategies to increase equity of access to services... [1].

This mandate exemplifies the expectations of political leaders for assuring that public funds are allocated according to need and in ways that support the goals of specific public health programs. Developing a formula that fulfills this mandate would be daunting and raises multiple questions: are timely and accurate data available that reflect the various dimensions of this mandate, how should specific indicators be combined and weighted mathematically, how should inequities in resource use or equity in access to services be defined? If the mix of considerations that shape allocations is too complex or nuanced, then attempting to allocate funds using a formula may not be the optimal approach; and inviting jurisdictions to submit funding requests and justifications that are reviewed by an independent panel of experts may be preferable. State and national public health agencies in the United States employ a combination of these two strategies to allocate program funds [2].

Controversy and debate can arise whenever public health agencies determine how finite program funds should be allocated among constituent jurisdictions, given the potential for "losers and winners" whenever alternative allocation strategies are considered. Under formula-based methods, the proportion of available resources allocated to each jurisdiction is determined by a mathematical calculation. Under competitive methods, allocations are based on expert review of applications. Competitive awards may be based on a *de facto *hybrid of qualitative and formulaic indicators, such as quantitative measures of morbidity or mortality used by applicants to justify proposed budgets. The merits of these formula-based versus competitive allocation approaches mirror one another. Formula-based allocations are generally simpler and more transparent, while competitive allocations are better geared to considering the complexities of local circumstances. While the allocation of huge sums is at stake when national and state-level public health programs are considered, there has been relatively little formal evaluation of formula allocation methods used by federal public health programs [2], with the notable exception of the Ryan White HIV CARE program, which has been the subject of an in-depth assessment by the Institute of Medicine and multiple reviews by the Government Accountability Office and others [3-7]. The purpose of this article is to inform debates regarding the use of formula-based funding methods.

## Discussion

### National Academy of Sciences Panel on Formula Allocations

Formula-based funding is used widely by multiple federal government agencies in the United States. Prompted initially by controversies surrounding education program funding, in 2000, the National Academy of Sciences convened a Panel on Formula Allocations, which issued reports in 2001 and 2003 [8,9]. The panel reported that in fiscal years 1999 and 2000, the federal government distributed over $250 billion using funding formulas. Medicaid, a healthcare insurance program for those who meet certain need or disability criteria, accounted for nearly half of this spending; followed by highway construction, education, and various social service programs. The panel reviewed the elements of formulas, including data sources and statistical methods, as well as the advantages, limits, and unintended consequences of formula allocation methods. The panel concluded its deliberations by recommending that the federal government's Office of Management and Budget establish a standing "Committee on Formula Allocations" to develop "improved simulation and quality-control techniques for use in formula design" and to prepare a handbook for legislators and program managers "that would serve as an introduction to underlying concepts and practical considerations in the use of formula-based fund allocation." [9]. To date, no such office has been established [2].

The NAS panel observed that funding formulas range from simple to complex, with simple formulas involving a single data source and complex formulas involving multiple data sources and adjustments for differences in the cost of providing services or the availability of local resources [8]. Formulas typically include a minimum funding level guaranteed to all recipients, with or without an eligibility threshold, a funding ceiling, and a "hold-harmless" provision to blunt effects of random fluctuations in source data. The panel concluded that transparency and perceived fairness were major advantages of formula-based allocations. These advantages can be offset if the design and legislative approval of formulas is excessively influenced by advocates from particular jurisdictions, if data sources and calculations imperfectly reflect program goals, if the use of a data source leads to anomalies in how data are collected (especially when funded jurisdictions are responsible for collecting the data), if perceived fairness is undermined by substantial variations in "per capita" or "per eligible" funding due to guaranteed minimums or hold-harmless provisions, or if a lack of clear definitions and procedures creates an appearance of gaming to suit particular jurisdictions. The NAS panel did not substantially consider the potentially unique challenges inherent in funding public health programs aimed at disease prevention. For example, if disease counts are used as the basis for funding a prevention program, an area that is successful in preventing illness could lose funding, threatening its ability to sustain its success. A classic illustration of the phenomenon of program success leading to a decrease in morbidity and a decrease in funding is the erosion of the infrastructure for tuberculosis prevention and control that occurred in the United States in 1970's and early 1980's, only to be followed by a resurgence in tuberculosis in the late 1980s and early 1990s [10,11]. Recognition of this resurgence prompted renewed support for tuberculosis programs, which enabled successful efforts to reverse unfavorable trends.[11]

### Ryan White HIV CARE program formula

Among formulas used by federal public health agencies in the United States, it is not surprising that the formulas used in the various "titles" or components of the Ryan White HIV CARE program have been subject to the greatest scrutiny, given both the role of political advocacy in shaping HIV care programs and controversies surrounding approaches to monitoring the HIV epidemic [7,12-14]. Evaluations of the Ryan White formula are extensively documented elsewhere [2-7], so we will touch on just a few points from prior reports that illustrate the challenges in designing a funding formula. Foremost is the limitation of the source data relative to program objectives, namely cases of AIDS reported to state health departments [4]. AIDS represents the late stage of HIV disease, but the program aims to serve people at all stages and to encourage early care. This limitation of the source data prompted Congress, when it reauthorized the Act in 2000, to mandate that HIV data be included in formula beginning in FY-2007 [4,5]. Moreover, program services are intended as a "safety net" for those who cannot otherwise afford care, due to limited or exhausted resources, yet people reported with AIDS include many who would not meet the program's financial eligibility criteria [4]. The formula originally used cumulative numbers of AIDS reports, which eventually included many who had died, prompting a shift to counts of people living with AIDS [4]. The "hold-harmless" provision embedded in this change contributed to substantial variations in "per case" funding, primarily for one geographic area, San Francisco, relative to others [4,5]. Without this provision, San Francisco, an area with a large and relatively early AIDS epidemic and thus a sizeable number of AIDS deaths, would have lost a substantial share of funding. With this provision, San Francisco receives a much higher "per case" allocation than other areas [4,5]. Costs for providing HIV care vary among regions of the country, but early efforts to incorporate a cost adjustment in the design of the formula bogged down in Congressional deliberations and were eventually abandoned [4]. Theoretically, a state that is highly successful in providing early HIV care could achieve a reduction in AIDS cases and a reduction in its proportional allocation as a result, although this particular consideration does not appear to have been a major concern in prior deliberations regarding the use of AIDS report data in the formula [2-6].

The Ryan White formula also illustrates the potential hazards of using data collected by grantees. States vary in the quality and completeness of their AIDS surveillance systems, and these variations are even greater for HIV reporting systems [4,6]. The latter is further complicated by debates surrounding the use of names in HIV reporting systems. While the vast majority of states have adopted name-based HIV reporting systems, a few adhere to the use of systems that employ anonymous coding procedures. To date, CDC has not endorsed the use of non-name systems, and thus states that use such systems are at risk for losing a share of funding, as HIV data are phased into use in the Ryan White formula [6]. Concern about the proposed impact of the pending inclusion of HIV report data in the formula is one reason why the National Alliance of State and Territorial AIDS Directors has proposed delaying the passage of the Ryan White reauthorization bill, pending further assessment of the impact of this and other proposed changes to the program [15].

**Figure 1:**
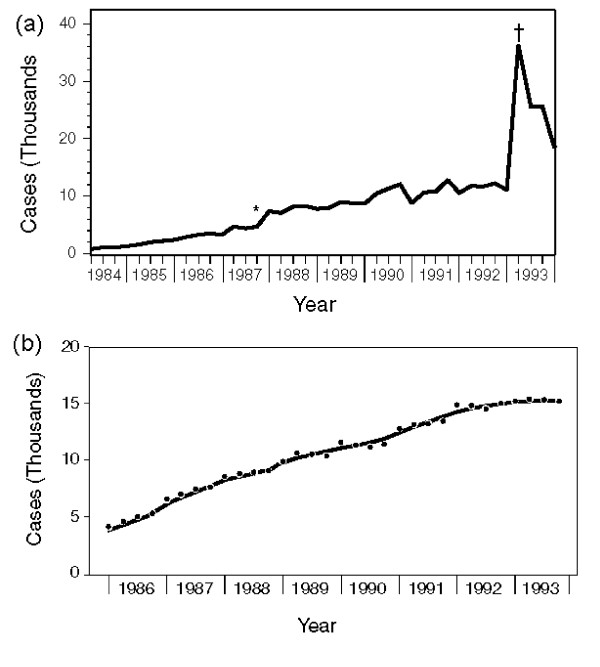
a – AIDS cases by date of report, United States, 1984–1993 Legend: Number of AIDS cases by quarter year of report, United States, 1984–1993. AIDS surveillance criteria were modified in 1987[*] and 1993[†]). Source: Centers for Disease Control and Prevention.[15] b – AIDS cases by date of diagnosis, United States, 1986-1993 Estimated AIDS-opportunistic illness incidence (represents an estimate of AIDS trends if the surveillance definition had not been revised in 1993), adjusted for delays in reporting, by quarter year of diagnosis, United States, 1986-1993. Source: Centers for Disease Control and Prevention.[17]

The use of AIDS surveillance data in the Ryan White formula provides an example of the way that funding formulas can affect source data. On January 1,1993, CDC implemented an expansion of AIDS surveillance criteria. This change had been deliberated for several years, and states anticipated its implementation. As a result, there was a huge spike in reported AIDS cases in early 1993, when examined by date cases were reported [16], which reflected the effect of the definition change and reporting incentives of the Ryan White program, not underlying trends in the epidemic (Figure 1a). This also led CDC to implement complex statistical adjustments to describe trends in AIDS by date of diagnosis had the definition not changed (Figure 1b) [16,17]. Lastly, the shift from cumulative numbers of AIDS reports, which included people who had died, to numbers of people living with AIDS [4], required consideration of the potential impact on the reporting of AIDS deaths. AIDS case reporting typically occurs in multiple steps, including an initial report followed by updates as additional information becomes available about subsequent opportunistic illnesses or death. A funding formula based on numbers of people living with AIDS could be a disincentive for states to assure that deaths are ascertained and reports updated. CDC averted this potential effect by using a national-level estimate of AIDS death rates and applying that estimate to all state AIDS reports [18].

### Formulas are not as transparent or value-free as they appear

Transparency means that an observer can readily determine how an allocation for a particular area was determined using a formula. This is true, however, only if the formula is carefully documented and the documentation is publicly available. For example, in January 2006, the U.S. Department of Health and Human Services announced plans to disperse approximately $100 million to "accelerate state and local pandemic influenza preparedness efforts" using a formula-based method. A press release described the formula in the following manner:

"...grants will be awarded to all 50 states, 7 territories, the Commonwealth of Puerto Rico, and the District of Columbia. Each state will receive a minimum of $500,000, with additional allocation of funds by population. In addition to the state grants, funds are being awarded to New York City, Chicago and Los Angeles County." [19].

No further information was released about the specifics of the formula, although a table accompanied the press release showing how much money each state, territory, commonwealth, and separately-funded city or county would receive [19]. From this information alone, it is not possible to re-create the reported allocations because reports about the formula do not specify 1) the source of census data, e.g., 2000 census data or inter-census estimates, with the latter being available on the Bureau of Census Internet site for more recent years for states than for other areas), 2) whether state calculations include or exclude the populations of separately-funded cities, although the latter can be presumed based on approximate calculations with available data, or 3) procedures used to define population-based funding provided to territories. Moreover, no rationale is provided for how either state or territorial baseline levels were determined.

**Figure 2:**
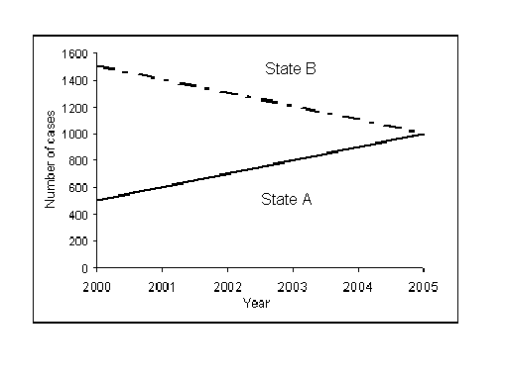
Trends in hypothetical disease for State A and State B, 2000-2005. Number of cases of a hypothetical disease reported in two states by year of report. These data are to be used in a formula calculation to allocate public health program funds between States A and B beginning in January 2007.

The perception of fairness of formula-based funding is based in part on the presumption that formulas represent an objective and evidence-based approach to resource allocation. While alluring, this perception can obscure the value-based judgments that necessarily underlie the design of any formula. This is illustrated by the example in Figure 2, which shows trends in incident case reports for a hypothetical disease for two states from 2000 to 2005. In this example, both states have the same number of incident cases in 2005 but arrived at this point via a decrease in one state and an increase in the other. These data are to be used in mid -year 2006 to fund a public health program that will begin January 2007. If a formula were based on the number of cases in 2005, the most recent calendar year for which full-year data are available when the funding decision is being made, each state would receive half of available funds. If the formula were based on the average number of cases for 2000–2005, the split would be approximately 40% for State A and 60% for State B, even though a plausible assumption is that by 2007 State A would have more cases than State B if recent trends predict future near-term trends. The allocation between states may differ more if it were based on prevalence rather than incidence or if projections in incidence or prevalence were considered. The relevance of these options for formula design would also differ if the program in question provided treatment services for people with the disease versus prevention services.

### Characteristics of national public health program formulas in the United States

**Figure 3:**
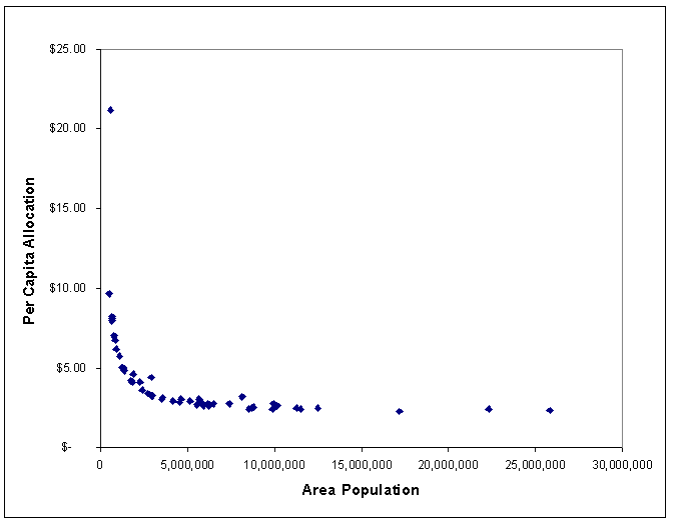
Per capita allocation, Public Health Emergency Preparedness Funding Program, United States, 2005. For the budget period August 31, 2005 – August 30, 2006, the Centers for Disease Control and Prevention awarded $809,956,000 to states and territories using the following allocation formula: "Each State awardee and Puerto Rico will receive a base amount of $3.91 million, plus an amount equal to its proportional share of the national population as reflected in the U.S. Census estimates for July 1, 2003. The District of Columbia will receive a base amount of $10 million and New York City, Los Angles County, and Chicago will continue to receive a base amount of $5 million."[21] The graph shows the resulting per capita allocation for states and separately funded cities/counties, by the state or city/county population.

In addition to the Ryan White HIV CARE program, major national programs from the United States Centers for Disease Control and Prevention and Health Resources Services Administration allocate hundreds of millions of dollars among states, territories, and selected urban areas using relatively simple formulas, based on either census counts, historical funding precedents, or a combination of these approaches, without adjustments for either the availability of local resources or inter-state variations in the cost of delivering services [2]. These include programs for bioterrorism and public health emergency preparedness, maternal and child health services, and a flexible "block grant" program that allows states to address health problems not addressed by more categorical programs. In addition to formula-allocated funds, each of these programs sets aside varying proportions of funds for programs designed to meet specific needs or to allow for innovation, and these set-aside funds may be awarded competitively or by funding formulas [2]. Because each also includes an explicit or *de facto *guaranteed minimum level of funding for each jurisdictions, there are substantial differences in per capita funding levels, as illustrated in Figure 3 for the bioterrorism and emergency preparedness program.

### Considerations for future research and evaluation in the design and use of funding formulas

Public health systems research in general and research into optimal public health financing strategies in particular are under-developed areas of inquiry and evaluation [20]. As a result, little practical and evidence-based guidance is available to inform decisions by public health managers regarding whether to allocate funds using a formula-based or competitive approach, or if the formula approach is selected, regarding optimal methods for constructing allocation formulas.

In addition to research that focuses on the utility of specific attributes of funding formulas, attention should also be focused on questions of how the selection of formula options reflects or suits different values or perspectives and on the ways that formulas can be or have been manipulated to suit the interests of particular jurisdictions. There are also underlying ethical questions that merit exploration, such as the weighing the importance of achieving comparable levels of per capita or per case funding (e.g., equity) versus recognizing that different levels of funding may be needed in different areas to achieve comparable results (e.g., equivalency). The recommendation of the National Academy of Sciences panel to develop a guide for those contemplating the use of formula allocations should be considered specifically for public health [8,9]. Lastly, there may be value in looking beyond the impact of formulas as part of individual programs. For example, how is the overall quality of life or the health of a state affected by the mix of formula-based federal public health programs? In the meantime, those who use formula-based strategies to allocate funds should be familiar with the advantages, limitations, and nuances of this approach.

## Summary

Public health agencies in the United States often allocate program funds among constituent jurisdictions using either formula-based or competitive methods. Formula-based methods may be simpler or more transparent than competitive allocations, but this appearance may be deceptive and mask underlying values or priorities that shape the selection of formula data sources or calculation methods. The design and use of funding formulas in public health represents an under-developed area of applied, practice-based research. As a result, little evidence-based guidance is available to those who seek to use formula-based allocation methods. Users of this approach should be familiar with its advantages, limits, and nuances.

## Competing interests

The author(s) declare that they have no competing interests.

## Authors' contributions

JB served as lead author of this report. DH contributed substantially to designing the report and provided review and feedback at each stage in its development.

## Pre-publication history

The pre-publication history for this paper can be accessed here:


